# New Drugs. Appliances. And Things Medical

**Published:** 1892-11-26

**Authors:** 


### NEW DRUGS, APPLIANCES, AND THINGS MEDICAL.

[All preparations, appliances, novelties, etc., of which a notice is desired, should be sent for the Editor, to care of The Manager, 140, Strand, London, W.C.]

### WHOLESOME BREAD.

Messrs. Bonthron, 106, Regent Street, have supplied us with samples of some very excellent bread, which we are pleased to introduce to our readers, many of whom may be glad to know where to procure excellently made and nutritious bread. We have tried both the brown and the white varieties sent to us, and find each excellent in their way. Both are made of standard whole-meal, patent dietetic flour, under a system invented by Mr. H. W. Hart, and manufactured by E. Bonthron. These breads retain a larger amount of nutriment, and are sweeter and more agreeable to the taste than most kinds we are acquainted with. The brown bread, whilst retaining the flavour which makes such bread preferable to many, is made of the finest flour procurable, and is, therefore, more digestible than the coarser kind usually used. A sweet ferment process is adopted, which is also an advantage. The brown meal used by Messrs. Bonthron we found to serve excellently for scone making.

### A NEW COCOA.

Messrs. Grootes' Dutch Cocoa Powder, one of the latest cocoas brought under our notice, deserves very high commendation. It is absolutely pure, highly concentrated, and agreeable in flavour. It is not expensive, and in use will be found economical, a small quantity going a long way. This cocoa enters a field we could have imagined already filled, but there are always those to be found in search of novelties, and those who are not content with our excellent home produce will do well to try that of Messrs. Grootes' manufacture.

